# Proton Conduction in Gly-X (X = Ser, Ser-Gly-Ser) and GS50

**DOI:** 10.3390/bioengineering10101223

**Published:** 2023-10-19

**Authors:** Hitoki Semizo, Ryusei Yabu, Yamato Ohgishi, Haruka Kai, Hitoshi Nishimura, Yasumitsu Matsuo

**Affiliations:** Faculty of Science & Engineering, Setsunan University, Ikeda-Nakamachi, Neyagawa 572-8508, Japan; 22m912yr@edu.setsunan.ac.jp (R.Y.); 23m910oy@edu.setsunan.ac.jp (Y.O.); h-kai@aitec-j.com (H.K.); nishimura@lif.setsunan.ac.jp (H.N.)

**Keywords:** biomaterial, amino acid, genetic recombination

## Abstract

In recent years, the use of biomaterials has been required from the viewpoint of biocompatibility of electronic devices. In this study, the proton conductivity of Glycyl-L-serine (Gly-Ser) was investigated to clarify the relationship between hydration and proton conduction in peptides. From the crystal and conductivity data, it was inferred that the proton conductivity in hydrated Gly-Ser crystals is caused by the cleavage and rearrangement of hydrogen bonds between hydration shells formed by hydrogen bonds between amino acids and water molecules. Moreover, a staircase-like change in proton conduction with hydration was observed at *n* = 0.3 and 0.5. These results indicate that proton transport in Gly-Ser is realized by hydration water. In addition, we also found that hydration of GSGS and GS50 can achieve proton conduction of Gly-Ser tetrameric GSGS and GS50 containing repeating sequences. The proton conductivity at *n* = 0.3 is due to percolation by the formation of proton-conducting pathways. In addition to these results, we found that proton conductivity at GS50 is realized by the diffusion constant of 3.21 × 10^−8^ cm^2^/s at GS50.

## 1. Introduction

As is well known, hydrogen fuel cells, which produce water through the reaction of hydrogen and oxygen, are currently attracting attention as a next-generation energy source. Hydrogen fuel cells are fuel cells that operate at low temperatures, and they are widely used as power sources for mobile devices and automobiles. Eroglu et al. report a mobile renewable home that utilizes a hybrid power system of solar, wind, and fuel cells [1]. Wang et al. describe recent advances in key materials and components for proton exchange membrane fuel cells in automotive applications [2]. Chan et al. report supported mixed-metal nanoparticles as electrocatalysts for low-temperature fuel cells [3]. Proton conductors are used as electrolytes in hydrogen fuel cells, and the development of inexpensive and environment-friendly proton conductors is desired. Tissue-derived biomaterials, such as nucleic acids, sugars, and proteins produced by living organisms, are inexpensive, abundant in nature, and environmentally friendly. In recent years, biomaterials have been applied to the energy field, in addition to the medical field. Chao et al. reported an oxide electric double-layer transistor gated with a chitosan-based biopolymer electrolyte [4]. Lai et al. synthesized a new bio-dielectric elastomer with a large working strain at a low electric field by free radical redox emulsion polymerization from di-n-butyl itaconate and isoprene. [5]. Furthermore, Yumusak et al. describe a bioorganic field effect transistor based on a crosslinked deoxyribonucleic acid (DNA) gate dielectric [6]. Collagen, a biological substance, is abundant in fish scales and other waste products, while cellulose is found in the cell walls of plant cells. Chitosan is also found in crab and shrimp shells and wood chips. These biomaterials have been suggested to have proton-conducting properties. [7,8,9]. It has also been reported that these biomaterials can be used as electrolytes to fabricate fuel cells [10,11,12]. Thus, biomaterials are inexpensive, abundant in nature, and have many useful functions, and are therefore desired as next-generation materials. Recently, proteins, which are derived from living organisms, have been attracting attention because of their applicability to biodevices [13]. Glycine, a protein-building amino acid, has the simplest side chain [14,15,16]. Collagen is a protein that makes up skin and other tissues. Proline is a well-known amino acid that is abundant in collagen [17,18,19]. The hydration structure of many biomaterials is related to the proton conduction mechanism, but the details have not been elucidated. This is the biggest challenge in the application of proteins to biodevices. Recently, a direct study of the relationship between proton conduction and hydration from the crystal structure of amino acid dipeptides reported the proton conductivity of glycylproline (Gly-Pro), which is abundant in collagen [20]. There, it is described that the proton conductivity of hydrated Gly-Pro crystals is caused by the recombination of hydrogen bonds within/among the hydrated shells formed between water molecules and amino acids. Furthermore, the increase in proton conductivity at hydration number *n* ~ 0.15 is due to the formation of a percolation network, suggesting that the anisotropy in proton conductivity is due to the arrangement of water molecules along the *c*-axis. Higher proton conductors are required when trying to apply peptides to energy devices. It seems that proton conduction in biological materials is achieved by hydration, and peptides with hydrophilic side chains are thought to achieve higher proton conduction. Serine, which is abundant in major proteins, has an OH group and is used in many fields. Panatier et al. have shown that glial-derived D-serine regulates NMDA receptor activity and synaptic memory [21]. Horn et al. describe D-serine as a neurotransmitter, as well as its role in brain development and disease [22]. Castillo et al. reported that D-serine, released by brainstem astrocytes, modulates respiratory responses to CO_2_ levels [23]. Thus, serine has various functions, depending on the OH group on its side chain; the OH group is hydrophilic and is known to achieve hydration, which is important for the proton conduction mechanism of biogenic substances. In this study, we prepared single crystals of glycyl serine (Gly-Ser), a peptide with an OH group on the side chain, and investigated the relationship between proton conduction and crystal structure.

In addition, understanding the relationship between proton conduction and crystal structure in Gly-X, Gly-Ser-Gly-Ser (GSGS) is essential as an initial step in proton-conducting peptide membranes; based on the Gly-Ser and GSGS results, the relationship between peptide length and proton conduction was investigated. Artificial proteins that further lengthen GSGS would greatly advance its application in devices. Oskolkov et al. describe how small modifications of the peptide sequence affect its ability to deliver to cells [24]. Ezzat et al. report novel cell-penetrating peptides for oligonucleotide delivery [25]. Chaloin et al. designed carrier peptide-oligonucleotide conjugate with rapid membrane translocation and nuclear localization properties [26]. However, when chemically synthesizing a protein, the cost depends on the length of its amino acids. Artificial proteins produced by genetic recombination using microorganisms are mainly applied in the medical field because they are inexpensive and biocompatible. Chen et al. report a protein-based artificial retina [27]. Petka et al. use recombinant DNA methods to create engineered proteins that undergo reversible gelation in response to changes in pH or temperature [28]. Gogurla et al. describe self-powering artificial skin made of artificial silk protein hydrogel [29]. Designing and creating an artificial protein that becomes a proton conductor by hydration can be an essential finding that greatly advances the application of proteins to devices. Therefore, we created and evaluated an artificial protein (GS50) containing 50 Gly-Ser repeats using genetic recombination technology with Escherichia coli. Therefore, we have also investigated proton conductivity in GSGS and GS50.

## 2. Materials and Methods

**Figure 1:**
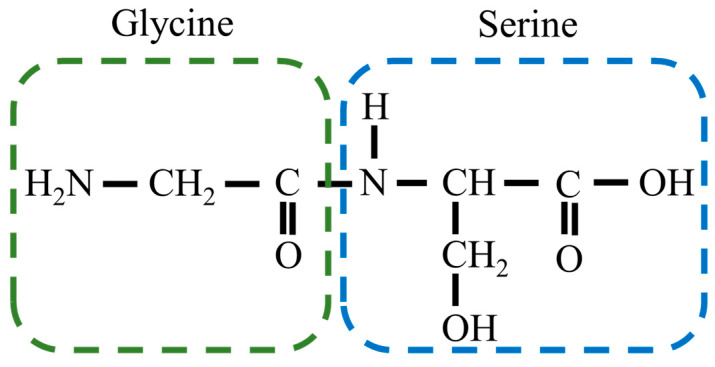
A single molecule of Gly-Ser unit.

**Figure 2:**
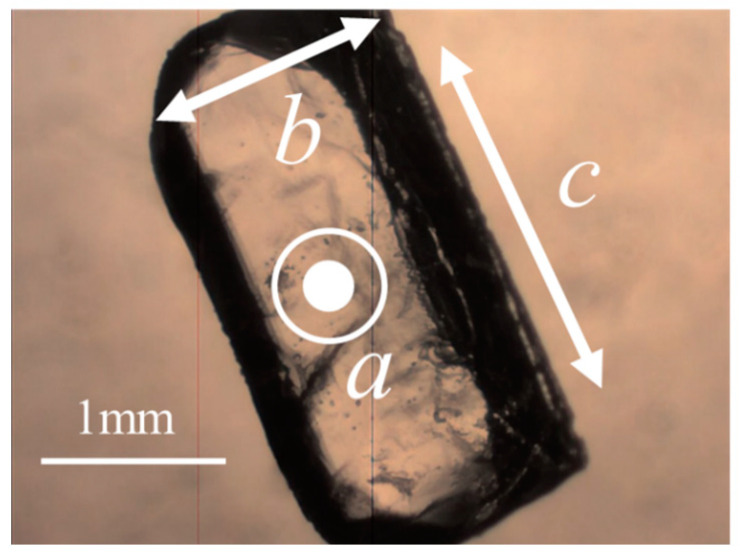
Measurement direction of the crystallized sample. *a*–*c* indicate the axial direction of the crystal.

**Figure 3:**
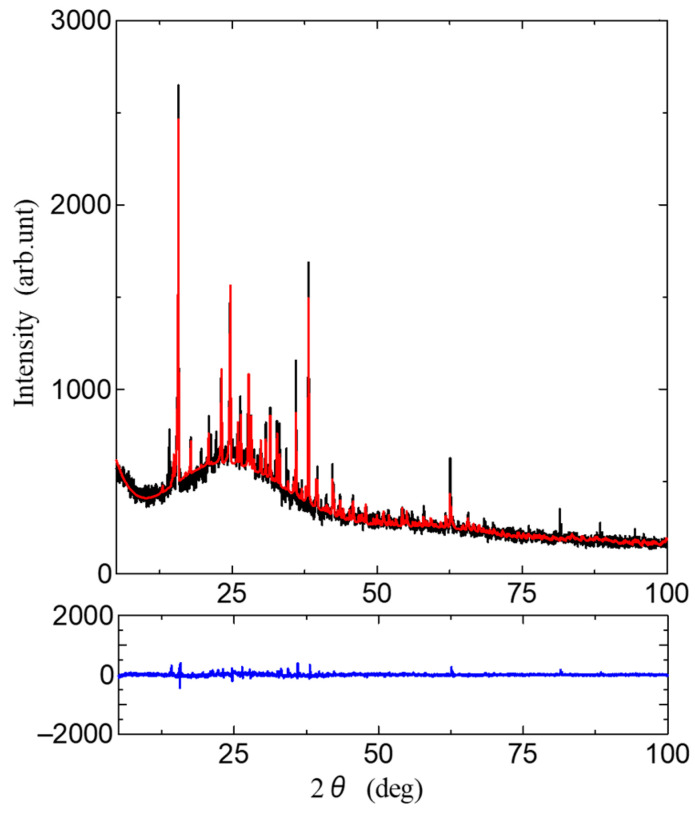
The XRD pattern of Gly-Ser. The blue line represents the difference between calculated and empirical intensities.

**Figure 4:**
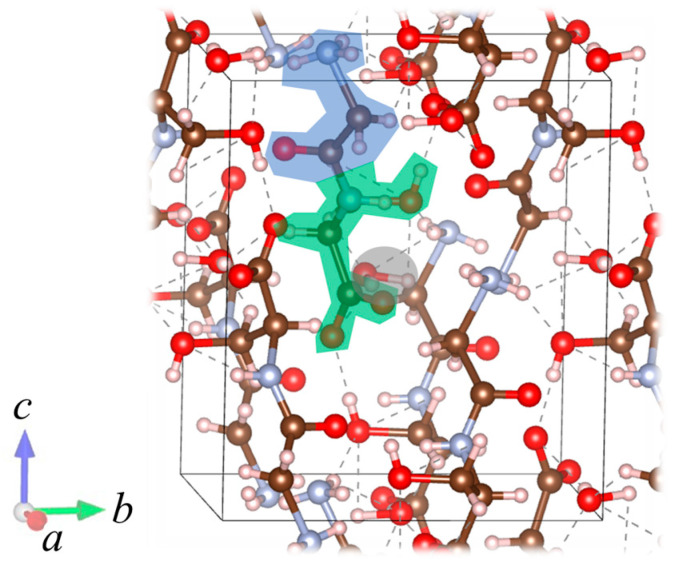
Determined Gly-Ser hydration structure. Green, blue, and gray represent the glycine, serine, and hydrated water molecules. *a*–*c* indicate the axial direction of the crystal.

Figure 1 shows a single molecule of Gly-Ser units. Crystals were obtained by heating a supersaturated aqueous solution of 0.1 g Gly-Ser powder with 200 μL of water to 30 °C and allowing it to stand for about 3 days. Figure 2 shows a Gly-Ser crystal. Proton conductivity was measured by an AC impedance method using electrodes attached to Gly-Ser peptide crystals. In this study, proton conductivity measurements were performed for each of the crystal axes (*a*-, *b*-, and *c*-axes). An LCR meter (E4980A, Agilent Technologies, CA, USA) was used for the conductivity measurements. The hydration structure of Gly-Ser was determined by an X-ray diffractometer (LabX XRD-6100, Shi-Madzu, Kyoto, Japan); X-ray diffraction measurements were performed in reflection mode using Cu-Kα radiation (40 kV, 30 mA) and a spectrometer. XRD measurements were taken at 0.02° per second (2*θ*). Figure 3 shows the XRD pattern of Gly-Ser. The structure of the Gly-Ser dipeptide obtained by Görbitz was used as an initial parameter to analyze the results of hydrated Gly-Ser [30]. As shown in Figure 3, sharp Bragg peaks were observed in the XRD pattern of the Gly-Ser crystal. This means that the sample has excellent crystallinity. The parameter *S*, which represents the reliability (goodness of fit), was 1.56. Rietveld analysis was performed on these results, and the obtained Gly-Ser structure is shown in Figure 4. The hydration structure indicates that many sites can form hydrogen bonds through water molecules. Furthermore, Table 1 shows the structural parameters of Gly-Ser obtained by this analysis.

**Figure 5:**
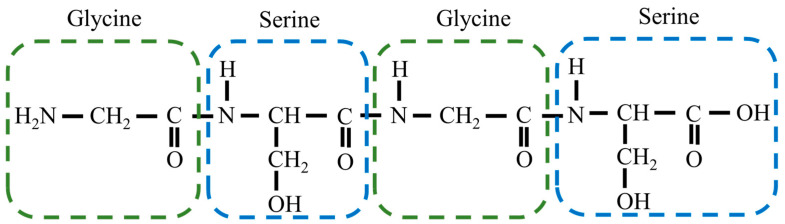
GSGS structure used in the present work.

Figure 5 shows a unit composed of GSGS. GSGS thin films were obtained by dissolving 0.4 mg of GSGS powder in 2 μL of water, dropping it onto a substrate, and drying it. Electrodes were attached to the GSGS thin film, and proton conductivity was measured by the AC impedance method.

**Figure 6:**
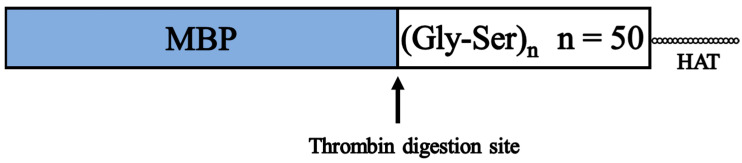
Schematic diagram of the engineered protein.

**Figure 7:**
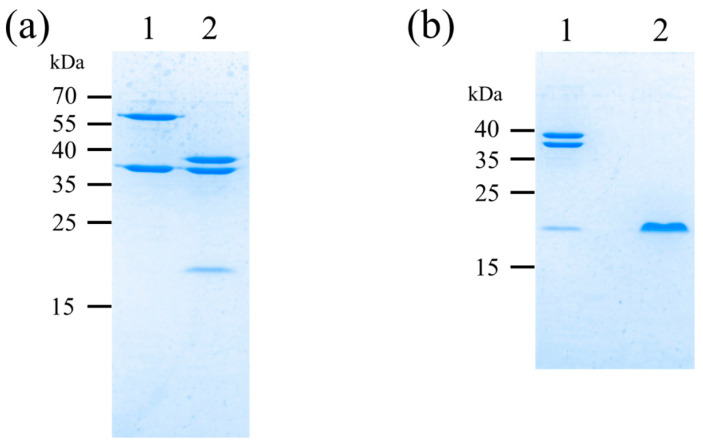
SDS-PAGE of expressed and purified GS50 (**a**) before and after thrombin digestion (1 = before thrombin digestion, 2 = after thrombin digestion) and (**b**) before and after purification of digested GS50 with Co-resin beads (1 = before purification with Co-resin beads, 2 = after purification with Co-resin beads).

Figure 6 shows a schematic of the designed artificial protein. Maltose-binding protein (MBP) is used for protein expression and purification. Pryor et al. achieved high expression of soluble proteins in E. coli by using a His6-tag and MBP double-affinity fusion system [31]. Riggs also describes a procedure for subcloning the sequence encoding the protein of interest into a MBP vector and then expressing and purifying the fusion protein from the cytoplasm [32]. Histidine affinity tags (HAT) are known for their less aggregation-prone protein aggregation characteristics compared to His tags; Chaga et al. researched the purification of recombinant proteins on cobalt (II)-carboxymethylaspartate-bridged agarose, incorporating a natural poly-histidine affinity tag (HAT) into the recombinant protein to facilitate expression and purification [33]. Sasaki et al. selected clones expressing HAT Der p1 and Der f4 proteins and purified the expressed recombinant proteins with HiTrap TALON crude [34]. To facilitate expression and purification, the protein is tagged with MBP at the N-terminus and HAT at the C-terminus; MBP and HAT can be purified with amylose resin and cobalt resin, respectively. We also used pET32a as the vector and BL21 (DE3) pLysS as the competent cell. After expression, MBP was removed by thrombin digestion and HAT purification to create a protein (GS50), in which more than 80% of the protein was composed of Gly-Ser. After culturing E. coli, IPTG was added to a final concentration of 0.5 mM to induce expression. After the cells were collected and crushed, the protein shown in Figure 6 was purified using an amylose resin column. In this case, thrombin digestion was inserted between MBP, and GS50 was used for expression. Thereby, the purified protein was thrombin digested, and GS50 was purified using cobalt resin beads. Figure 7a shows the SDS-PAGE results of the samples before and after thrombin digestion. Lane 1 shows the results before thrombin digestion, and lane 2 shows the results after digestion, indicating that the original protein was completely degraded into MBP and GS50. Figure 7b shows the SDS-PAGE results of samples before and after purification of digested GS50 with Co-resin beads. Lane 1 shows the results before Co-resin purification, and lane 2 shows the results after purification. As shown, only GS50 with the HAT tag could be purified and concentrated, and the protein concentration after desalting was 0.1 mg/mL. The humidity dependence of the proton conductivity of GS50 was measured by dropping this aqueous GS50 solution onto a substrate, drying it, and thinning it.

## 3. Results

**Figure 8:**
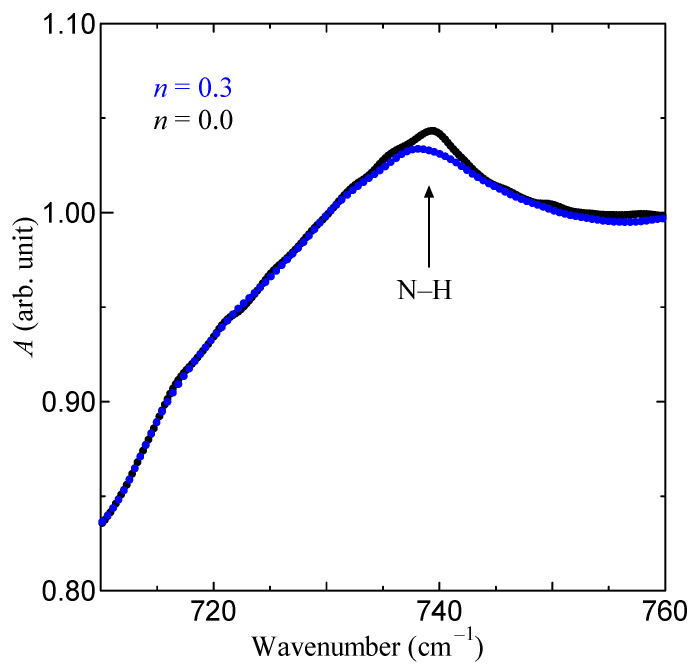
FT-IR spectra of Gly-Ser under each *n*.

In order to confirm the existence of the binding between water and Gly-Ser molecules, we carried out IR measurements using the FT-IR spectrophotometer (Nicoret iS5: Thermo Fisher Scientific Inc, Massachusetts, USA). Figure 8 shows the FT-IR spectra of Gly-Ser under each *n*. As shown in Figure 8, the peak intensities at 740 cm^−1^ at *n* = 0.3 were decreased. The *n* represents the number of water molecules per Gly-Ser molecule, and the amide V band at 740 cm^−1^ indicates the presence of N-H refractive vibrations [35]. Thus, the reduction of the peaks at 740 cm^−1^ means that water molecules are suppressing N-H motions. This indicates that the hydrated water molecule binds to the amide nitrogen of Gly-Ser.

**Figure 9:**
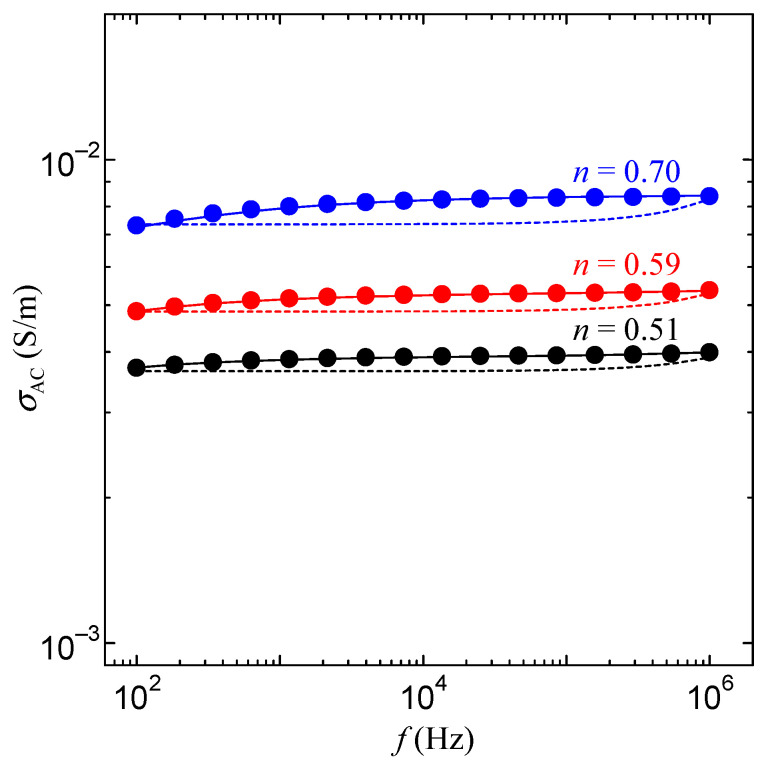
Conductivity properties of Gly-Ser crystals at each hydration condition.

Figure 9 shows the conduction properties of Gly-Ser crystals under each hydration condition. In Figure 9, the *σ*_AC_ increases with higher frequencies. The *σ*_AC_ increases with hydration number *n*, indicating that the Gly-Ser crystal achieves proton conduction by hydration water. In addition, parameters related to proton conduction were clarified. First, the proton conductivity obtained by AC impedance measurement can be described by a parallel equivalent circuit of resistance *R* and capacitance *C*, as in Equation (1):

*σ*_AC_ = *σ*_0_ + *ωε*_0_*ε*″, (1)

where *σ*_0_ is the DC conductivity, *ω* is the angular frequency, *ε*_0_ is the dielectric constant in vacuum, and *ε*″ is the imaginary component of the dielectric constant. The frequency dependence of *σ*_AC_ calculated by Equation (1), and shown by the dashed line in Figure 9, is not consistent with the measured value. This result indicates that there is another component, other than the simple parallel equivalent circuit of *R* and *C* in the Gly-Ser crystal sample. It is well known that biopolymers exhibit non-debyelectric relaxation due to α-relaxation of the electric dipole moment in the hydration shell formed by water molecules and side chains [20]. Therefore, hydrated Gly-Ser crystals should be considered to contain dielectric dispersion. The AC conductivity including dielectric dispersion is expressed by Equation (2):

(2) $$\sigma_{AC}=\sigma_{0}-Im\left[\omega \varepsilon_{0}\varepsilon_{\infty }+\frac{\omega \varepsilon_{0}\left(\varepsilon_{s}-\varepsilon_{\infty }\right)}{1+{\left(j\omega \tau \right)}^{\beta }}\right]\;=\sigma_{0}+\frac{\omega \varepsilon_{0}\left(\varepsilon_{s}-\varepsilon_{\infty }\right){\left(\omega \tau \right)}^{\beta }\sin\left(\frac{\pi }{2}\beta \right)}{{\left(1+{\left(\omega \tau \right)}^{\beta }\cos\left(\frac{\pi }{2}\beta \right)\right)}^{2}+{\left({\left(\omega \tau \right)}^{\beta }\sin\left(\frac{\pi }{2}\right)\right)}^{2}}$$

where *σ*_0_ is the DC conductivity, *ε*_s_ is the static dielectric constant of the Gly-Ser crystal, *ε*_∞_ is the high-frequency dielectric constant, *τ* is the relaxation time, and *β* is a parameter indicating the degree of multiple dielectric dispersion. *τ* represents the time for the dipole to flip and *β* represents the number of different types of relaxation times. Thus, the frequency dependence of *σ*_AC_ of hydrated Gly-Ser crystal with dielectric dispersion can be calculated using Equation (2). The results calculated using Equation (2) are shown as solid lines in Figure 9. The obtained fitting parameter values for each hydration condition are shown in Table 2. From Figure 9, the measured and calculated values of the frequency dependence are in good agreement. These results indicate that the DC proton conductivity, dielectric constant, and relaxation time values for Gly-Ser crystals can be obtained by using Equation (2).

**Figure 10:**
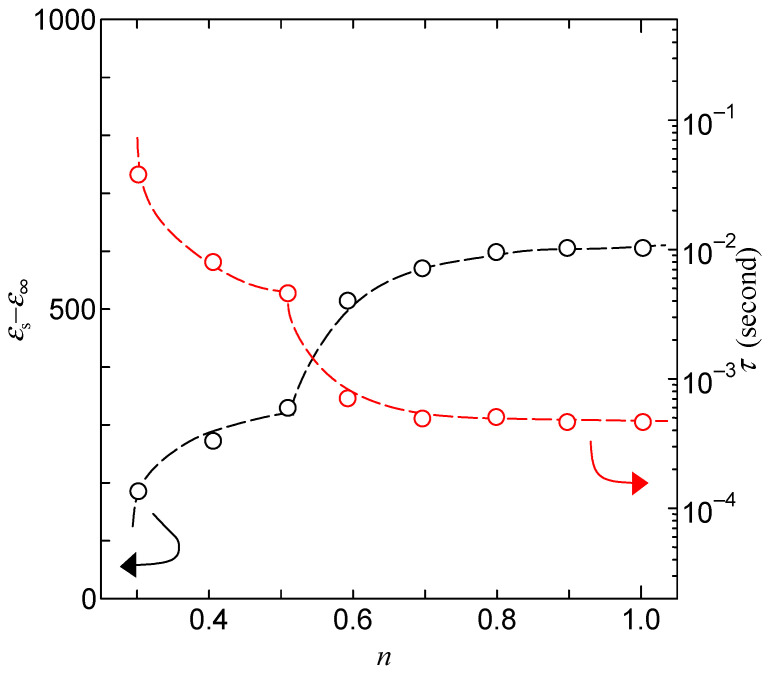
Dielectric constant and relaxation time of Gly-Ser at each hydration condition. Black represents *ε*_s_−*ε*_∞_ and red represents *τ*.

Figure 10 shows the dielectric constant and relaxation time of Gly-Ser at each hydration condition, obtained using Equation (2). From Figure 10, *ε*_s_−*ε*_∞_ in Gly-Ser crystals increases stepwise from *n* ~ 0.3 and *n* ~ 0.5. The increase in *ε*_s_−*ε*_∞_, as well as conductivity, is attributed to the formation of hydration shells by hydrated water molecules and side chains of Gly-Ser molecules. Thus, the dielectric dispersion observed in Gly-Ser crystals in response to AC electric fields is due to the flip-flop motion of dipole moments in the hydration shell. Figure 10 also shows the dependence of the dielectric relaxation time *τ* on the hydration number. From Figure 10, *τ* decreases with increasing *n*, in contrast to *ε*_s_−*ε*_∞_. The *n* represents the amount of water per Gly-Ser molecule (hydration number). The water content and hydration number of the samples used in the experiment were derived from the gravimetric measurements of the samples. Since the dielectric relaxation is due to the dipole motion of the side chain of the Gly-Ser molecule and the hydration shell bound to the water molecule, the decrease in *τ* is due to the faster flip-flop motion of the dipole movement as *n* increases. It is also noteworthy that, in Figure 10, a stepwise decrease in *τ* can be seen for *n* ~ 0.3 and *n* ~ 0.5. In Section 4, we will describe the proton conduction mechanism in hydrated Gly-Ser crystals with these important properties.

**Figure 11:**
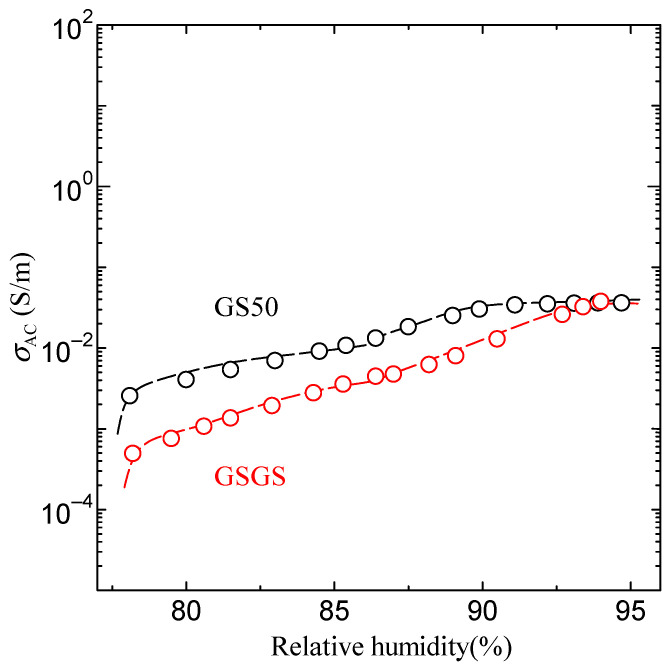
Humidity dependence of proton conductivity of GSGS and GS50. Black indicates GS50, red indicates GSGS.

In order to investigate the effect of the long chain of Gly-Ser peptide on proton conductivity, we have measured the proton conductivity in GSGS and GS50 films. Figure 11 shows the humidity dependence of proton conductivity at GSGS and GS50. As can be seen, the conductivity of GSGS and GS50 increases with increasing relative humidity. The conductivity properties of Gly-Ser, GSGS, and GS50 indicate a similar relationship between hydration and side chains in the Gly-Ser system. Therefore, the hydration number of GSGS and GS50 is estimated to be 0.5 from the gravimetric measurement of Gly-Ser at 87% humidity. It is also noted that the stepwise increase of proton conductivity is observed at around a relative humidity of 87%, corresponding to *n* = 0.5, in both GSGS and GS50 films. These results indicate that GSGS and GS50 achieve proton conduction through hydration with the same proton conductivity mechanism. In addition, Gly-Ser, GSGS, and GS50, all of which have OH groups on their side chains, exhibit almost the same high conductivity under hydration conditions.

**Figure 12:**
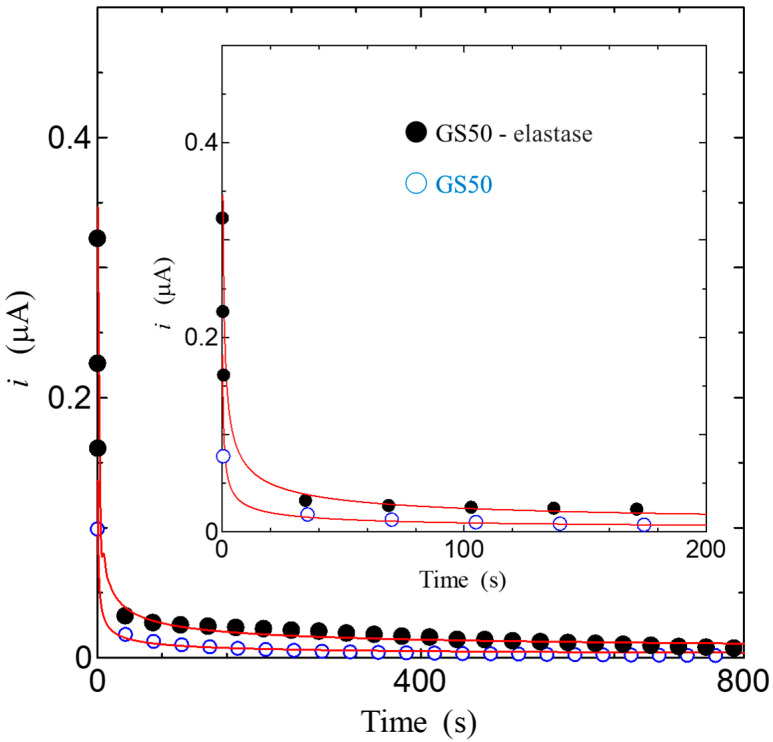
Time dependence of transient current after applying step voltage of 0.6 V. The inset shows the enlarged data.

In addition, the proton diffusion constant, which is an evaluation index of proton conductivity, is a constant that expresses the speed of diffusion of particles in a medium and is an essential index for application to devices. In the present work, we estimated the proton diffusion constant and carrier concentration using the Amperometry method with the enzyme [36]. The measurement system of the Amperometry method is very simple, consisting of a DC power supply that applies a step voltage, an ammeter that measures the current, and a computer that controls them. In this study, we used a regulated DC power supply (Keithley 2400) to apply a step voltage. The current was measured with a precision digital multi-meter (Keithley 2100). For the measurement, GS50 thin films were used, and gold electrodes were attached to them. Figure 12 shows the results of the time dependence of the transient current after the application of a step voltage of 0.6 V. In this measurement, a step voltage of 0.6 V was applied as the value at which the transient currents of GS50 and GS50-elastase complex coincide at *t→*∞. The transient current refers to a change in current value over time after a step voltage is applied, and the electrical characteristics of the sample can be revealed from the slope of the transient current. As shown in Figure 12, so-called “transient currents” can be observed in both GS50 and GS50-elastase complexes. The behavior of these transient currents is very similar, suggesting that the transient currents of GS50 and GS50-elastase complexes decay by the same mechanism. These results suggest the presence of currents generated by carriers produced in the GS50-elastase complex. Considering that the GS50 thin film is proton conductive upon hydration, as shown in Figure 12, it is suggested that the transient current is generated by the diffusion of protons in the GS50-elastase thin film.

Transient currents due to carrier diffusion have already been analyzed in various ways. One of these ways is by solving the diffusion equation. The diffusion of ions due to transient current is described by Fick’s first law, as in Equation (3):

(3) $$i=nFAD_{o}{\left(\frac{\partial C_{o}}{\partial x}\right)}_{x=0}$$

where *n* is the number of charges, *F* is Faraday’s constant (9.6485 × 10^4^ C/mol) for a charge per amount of electron mass, *A* is the electrode area, and *D*_o_ and *C*_o_ are the diffusion constant and carrier concentration, respectively. The symbol *x* represents the distance from the electrode interface to the bulk of the sample. When the initial condition *t* = 0, *C* = *C*_o_ when *x* ≥ 0, *C* = 0 when the boundary condition *x* = 0, and *C* → *C*_o_ when *x* → ∞, the carrier concentration gradient in this Equation can be obtained using the error function erf as follows:

(4) $$c(t,x)=C_{o}\mathrm{erf}\left(\frac{x}{2(D_{o}t{)}^{1/2}}\right)$$

Differentiating and substituting this into Equation (3) yields Equation (5), as follows:

(5) $$i=\frac{nFAD_{o}C_{o}}{(\pi D_{o}t{)}^{1/2}}$$

This Equation shows that the diffusion current (transient current) at a flat electrode is inversely proportional to the square root of time, called the Cottrell equation. The solid line in Figure 12 shows the results calculated by the Cottrell equation in Equation (5). As shown in the solid line in Figure 12, the transient current decreases at *t* ^−1/2^ for both the GS50 film and the GS50-elastase complex, following the Cottrell equation. The carrier concentration *C*_o_ and the diffusion constant *D*_o_ cannot be determined separately. However, the product of the two, *C*_o_*D*_o_^1/2^, can be obtained directly from experimental results. From equation (5), the *C*_o_*D*_o_^1/2^ of the GS50 membrane is 1.94 × 10^−9^, and the *C*_o_*D*_o_^1/2^ of the GS50-elastase complex is 4.71 × 10^−9^ mol/cm^2^∙s^−1/2^. In addition to this important value, the diffusion constant and carrier concentration in the system can be uniquely determined once the *C*_o_ or *D*_o_ values are obtained. As is well known, enzymes are catalysts and play a role in facilitating reactions, although the enzyme itself does not change between the starting and ending states of the reaction. The elastase used in this study cleaves amide bonds between amino acid molecules by hydrolysis. This result suggests that elastase, a known hydrolytic enzyme, is responsible for increasing the number of protons by generating new protons, which are carriers of GS50. In other words, given that elastase is to increase the number of protons, the increase in transient current in the GS50-elastase complex is not due to a change in diffusion constant, but rather to a change in carrier concentration. Therefore, assuming that the change in the diffusion constant due to elastase is negligible, the carrier concentration produced in GS50 by elastase can be roughly estimated from the measurements shown in Figure 12. The product of the carrier concentration and diffusion constant for GS50 and the GS50-elastase complex is given as the slope, *C*_o_g*D*_o_g^1/2^, and *C*_oe_*D*_oe_^1/2^, respectively. If the diffusion constant is *D*_og_ = *D*_oe_(= *D*_o_), the carrier concentration *C*_oe_ produced in the GS50-elastase complex is 2.4 times the carrier concentration *C*_og_ in the GS50 membrane. This result also suggests that *C*_og_, *C*_oe_, and *D*_og_ can be uniquely determined if another relationship between *C*_og_ and *C*_oe_ is obtained. We will further discuss this in the next section.

## 4. Discussion

**Figure 13:**
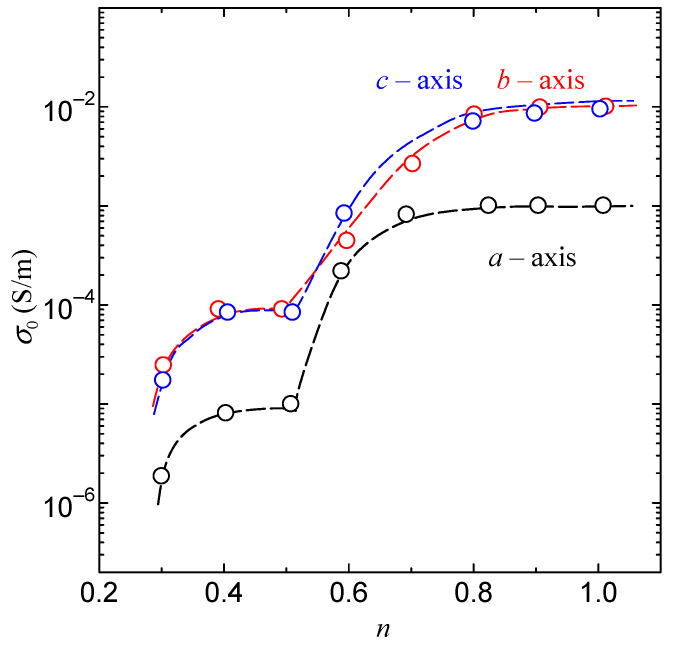
The conductivity of Gly-Ser crystals under each hydration condition.

**Figure 14:**
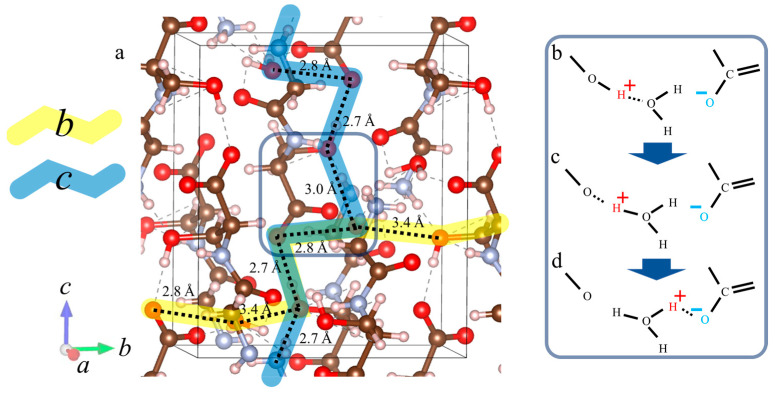
Hydration structure of the determined Gly-Ser crystal and detailed proton conduction mechanism. (**a**) Hydration structure of Gly-Ser crystal and proton conduction pathways. *a*–*c* indicate the axial direction of the crystal. Yellow and blue indicate proton conduction pathways along the *b*- and *c*-axis directions, respectively. (**b**) Hydrogen bonds are formed between the OH groups of Gly-Ser side chains and hydrated water molecules. (**c**) Recombination of the hydrogen bond forms an oxonium ion. (**d**) A hydrogen bond is formed between the oxonium ion and the oxygen of the adjacent carbonyl group.

In this study, we report the proton conductivity in hydrated Gly-Ser crystals and investigate the proton conduction mechanism from the crystal structure. The relationship between *n* and each parameter related to proton conduction in Gly-Ser crystals has already been introduced in Section 3. In this section, we discuss the relationship between hydration water and proton conduction mechanism in Gly-Ser crystals from micro and macro perspectives. Figure 13 shows that the conductivity in each axial direction in Gly-Ser crystals increases with increasing *n*. This result indicates that hydration water contributes to the increase in proton conduction in Gly-Ser crystals. In other words, the proton conductivity increases due to the formation of a hydration shell between the hydration water molecules and the side chains of the Gly-Ser molecule. Figure 14a shows the hydration structure of Gly-Ser obtained by Rietveld analysis of the XRD pattern in Figure 3. Note from Figure 14a that a hydration shell is formed consisting of hydrated water molecules and Gly-Ser hydroxyl groups, and that there are numerous hydrogen bonds within/between the hydration shells. In biomaterials such as Gly-Pro, it has been shown that proton conduction is realized by repeated cleavage and rearrangement of hydrogen bonds between hydrated water molecules and amino acid side chains [20]. From these facts, it is inferred that proton conduction in Gly-Ser crystals is realized by a recombination of hydrogen bonds within/between the hydration shell consisting of Gly-Ser side chains and hydrated water molecules. First, a hydrogen bond is formed between the hydroxyl group on the side chain of Gly-Ser and the hydrated water molecule (Figure 14b). Subsequent recombination of the hydrogen bond results in the formation of an oxonium ion from the water molecule (Figure 14c). Next, a hydrogen bond is formed between the oxonium ion and the adjacent oxygen molecule, recombination occurs, and the oxygen receives hydrogen (Figure 14d). As shown in Figure 14, proton conduction in hydrated Gly-Ser crystals is achieved by repeated hydrogen bond cleavage and recombination. The conductivity of Gly-Ser is 10~100 times lower than that of proton exchange membranes already in industrial applications. However, it can be improved by searching for amino acids with side chains that realize more hydrogen bonding networks, and by lengthening the chains.

Furthermore, from Figure 13, the proton conductivity along the *b-* and *c-*axes is particularly high. Figure 14a shows that the hydrated water molecules are located in the *b*-*c* plane. These results suggest that the high conductivity of Gly-Ser crystals along the *b*- and *c*-axes is due to two-dimensional conduction by hydrated water molecules arranged in the *b*-*c* plane. This result is reasonable, considering the proton conduction mechanism that accounts for the cleavage and rearrangement of hydrogen bonds formed between the hydroxyl group of the Gly-Ser side chain and the hydrated water molecule. The Gly-Pro is a one-dimensional conduction along the *c*-axis, whereas the Gly-Ser is a two-dimensional conduction in the *b*-*c* plane. It seems that the dimensionality of proton conduction is closely related to the conductivity of the peptide. It is also noteworthy that there is a stepwise increase in proton conductivity at *n* ~ 0.5 in Figure 13. In Figure 13, the proton conductivity increases stepwise. This is an important feature in clarifying the relationship between hydration and proton conduction in Gly-Ser crystals. In Figure 8, the frequency dependence calculated by Equation (2) is in good agreement with the measured values. In other words, the hydrated Gly-Ser crystal is shown to have dielectric relaxation. These results indicate that the dielectric relaxation observed in Gly-Ser is caused by the flip-flop motion of the electric dipole moment formed between the amino acid side chain and the hydration water molecule, the same mechanism as previously reported for Gly-Pro [20]. Thus, *τ* in Figure 9 has information about the rearrangement of hydrogen bonds within/between hydration shells in Gly-Ser. In Figure 10, *τ* decreases stepwise around *n* ~ 0.3 and *n* ~ 0.5. This result indicates a stepwise increase in the dipole flip-flop motion of the hydration shells near *n* ~ 0.3 and *n* ~ 0.5. Considering that the dipole moment is caused by the rearrangement of hydrogen bonds within/among hydrogen bond hydration shells, the stepwise decrease in *τ* around *n* ~ 0.3 and *n* ~ 0.5 is due to the faster rearrangement of hydrogen bonds around *n* ~ 0.3 and *n* ~ 0.5. This result is supported by the stepwise increase in proton conductivity with hydrogen bond rearrangement around *n* ~ 0.3 and *n* ~ 0.5 in Figure 8.

**Figure 15:**
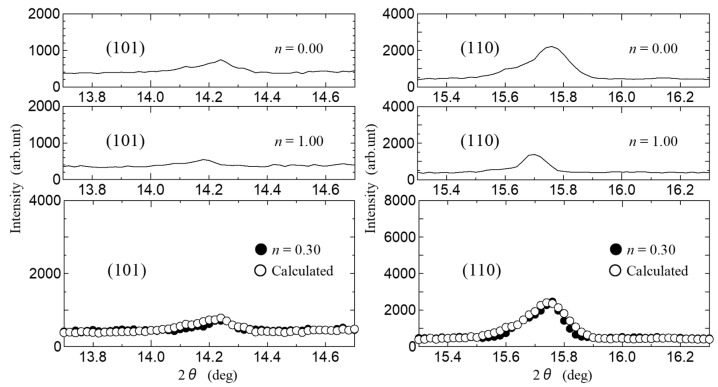
XRD pattern for the Miller indices (101) and (110). Calculated is for a 7:3 existence ratio of *n* = 0 and *n* = 1.0.

**Figure 16:**
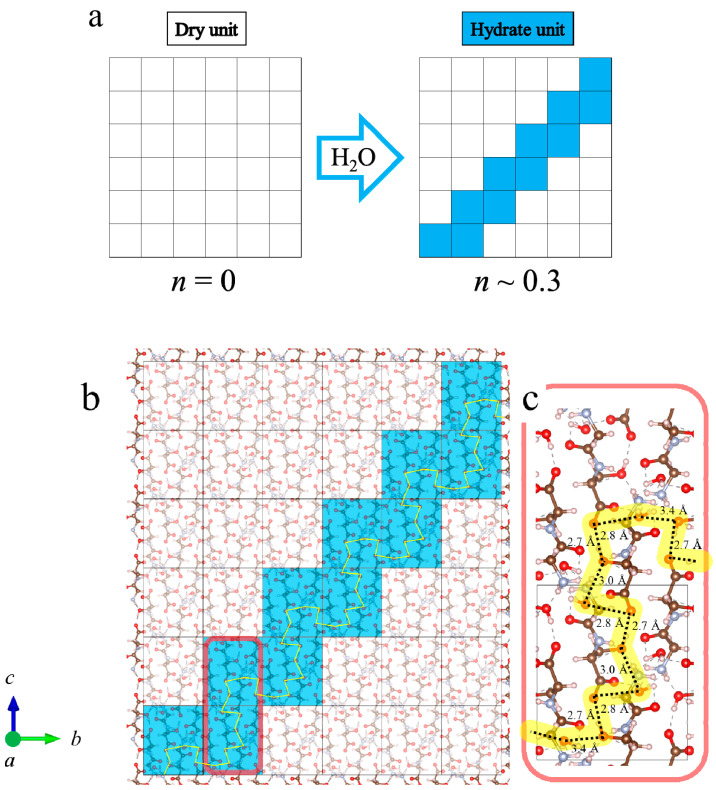
(**a**) Percolation model for proton conduction in Gly-Ser crystals. There are 144 Gly-Ser molecules and 44 water molecules, consistent with *n* ~ 0.3. *a*–*c* indicate the axial direction of the crystal. (**b**) Crystal structure of Gly-Ser. The hydration structure at *n* ~ 0.3 is shown, and the yellow color represents the proton-conducting pathway connected by hydrogen bonds in the hydration shell. (**c**) Magnified view of microscopic proton conduction.

Next, we discuss the increase in conductivity at *n* ~ 0.3, as shown in Figure 8, in terms of the Gly-Ser hydration structure revealed in this study. This result is noteworthy because it means that proton conduction pathways can be formed even with hydration numbers as low as *n* ~ 0.3. It has already been noted that, for many conductors, the rapid increase in conductivity is explained by the percolation model [20]. These results indicate that, for proton conduction to occur at low hydration conditions of *n* ~ 0.3, the formation of a conduction pathway for protons through the hydration shell is necessary. Therefore, the appearance of conductivity in the low hydration state is discussed using a percolation model. The shift of the diffraction peak indexed by the Miller index is important because it signifies the lattice change due to hydration in Gly-Ser. To demonstrate the presence of percolation effects in proton conduction, we focus on the diffraction peaks in the Gly-Ser crystal. Figure 15 shows the XRD patterns for the Miller indices (101) and (110) at *n* ~ 0.3 hydration. From Figure 15, the diffraction peaks change with *n*. For each hydration condition, the position of the diffraction peak changes due to the insertion of water molecules. Therefore, the crystal structure at *n* = 0.3 can be explained by the superposition of *n* = 0 and *n* = 1 diffraction peaks. Figure 15 shows that the XRD pattern for *n* = 0.3 is consistent with a 7:3 ratio superposition of *n* = 0 and *n* = 1 patterns. Since a crystal structure with *n* = 1 realizes proton conduction, proton conduction is realized at *n* ~ 0.3 if the hydrogen bonding network in the hydration shell is connected to the conduction pathway by percolation. Figure 16a is a percolation model for proton conduction in Gly-Ser crystals. It is worth noting that this is consistent with the phenomenon of increased conductivity under low hydration conditions in Figure 8. The 36 squares in Figure 16a represent 144 Gly-Ser molecules, with 44 water molecules inserted into them. Figure 16b shows the structure of the Gly-Ser molecule at *n* ~ 0.3. From this structure, proton conduction is achieved as a result of the formation of proton-conducting pathways. These results show that proton conduction is achieved by percolation at the said low hydration condition of *n* ~ 0.3. The present percolation shows that the stochastic connection of hydration shells in the Gly-Ser unit cell increases the conductivity. When the hydrated unit cell is a conductor and the anhydrous unit cell is a nonconductor, proton conduction is achieved with a relatively small number of hydrations, as the hydrated cells are connected (as in the model in Figure 16). This was first demonstrated by proton conductivity measurements and XRD results.

As can be seen in Figure 11 and Figure 12, the conductivity of GSGS and GS50 increases with increasing relative humidity. The results of the stepwise pattern have the same tendency, and the stepwise *n* can be estimated to be about 0.5, which is the same as that of Gly-Ser, suggesting that proton conduction has the same mechanism. This suggests that the conduction pathways of Gly-Ser, GSGS, and GS50 are linked by hydrogen bonds formed in the hydration shell, respectively.

**Figure 17:**
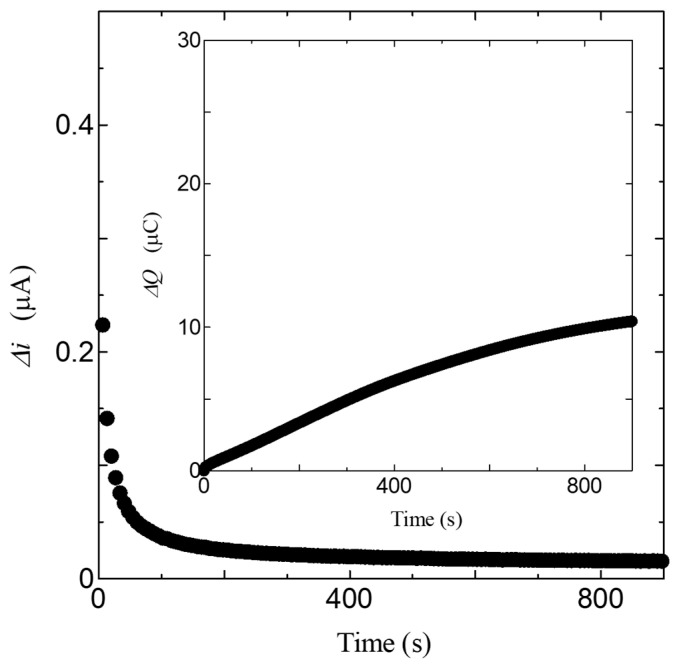
Time dependence of Δ*i.* Inset is a time dependence of Δ*Q*.

Finally, we discuss the results of transient current measurements on the GS50. From Figure 17, Δ*i*, the difference in transient current between the GS50 membrane and the GS50-elastase complex decreases rapidly with time. The Δ*i* on page 15 is analyzed because it results from proton production by the enzyme, and the total charge, Δ*Q*, is obtained from Δ*i*. Integrating the time dependence of Δ*Q* and Δ*i* yields the total amount of charge produced by the enzymatic reaction. As shown in Figure 17, the total charge nearly saturates at about 800 s. Therefore, the number of carriers generated in the GS50-elastase complex can be obtained by dividing the saturation charge by the charge factor. The difference in carrier concentration between the GS50 membrane and the GS50-elastase complex is 9.32 × 10^18^ cm^−3^, considering the volume of the GS50 membrane. *C*_og_, *C*_oe_, and *D*_o_ are estimated to be 6.51 × 10^18^ cm^−3^, 1.58 × 10^19^ cm^−3^, and 3.21 × 10^−8^ cm^2^/s, since the ratio of this value to the slope obtained in Section 3 is 2.4 times.

## 5. Conclusions

In this paper, we investigated the relationship between the proton conductivity of Gly-Ser crystals and the hydration structure in order to clarify the proton conductivity of Gly-Ser and the factors contributing to it. The results suggest that the proton conductivity of Gly-Ser crystals is realized by the rearrangement of hydrogen bonds between Gly-Ser side chains and hydrated water molecules, especially along the *b*- or *c*-axis. In addition, water molecules are inserted between the *b*-*c* planes from the Gly-Ser crystal structure. From these results, the proton conductivity in Gly-Ser is realized by the repeated cleavage and rearrangement of hydrogen bonds within/between the hydration shell consisting of Gly-Ser side chains and water molecules. AC impedance measurements of Gly-Ser crystals confirmed the presence of dielectric relaxation due to proton transfer in the hydration shells, with a stepwise increase in proton conductivity at *n* ~ 0.3 and *n* ~ 0.5. This result indicates that the rapid increase in proton conductivity at *n* ~ 0.3 and *n* ~ 0.5 is due to increased proton transfer within/between hydrated shells in Gly-Ser. The powder XRD pattern for *n* = 0.3 is consistent with a 7:3 ratio superposition of *n* = 0 and *n* = 1 patterns. The crystal structure of *n* = 1 achieves proton conduction, suggesting that the onset of proton conductivity at *n* ~ 0.3 is due to the percolation of hydrogen bonds in the hydration shell. Furthermore, we observed that both GSGS and GS50 show almost the same high conductivity under hydration conditions. Furthermore, proton conductivity increases around the relative humidity corresponding to *n* = 0.5 for both GSGS and GS50 films. These results indicate that proton conductivity in GSGS and GS50 films, which have a long chain of the Gly-Ser sequence, is also realized with the same proton conductivity mechanism in hydrated Gly-Ser crystal. These facts bring inexpensive and environmentally friendly bio-devices for the future.

## Figures and Tables

**Table 1:** Crystal data, Santa Clara, CA, USA.

Chemical formula	C_5_H_10_N_2_O_5_H_2_O
Chemical formula weight	198.15
Space group	P2_1_2_1_2_1_
*a* (Å)	7.266
*b* (Å)	9.120
*c* (Å)	10.60
V (Å^3^)	702.4
Z	4
Radiation type	Cu Kα

**Table 2:** Conductivity and dielectric relaxation parameters of Gly-Ser at each hydration condition.

*n*	*σ*_0_ (S/m)	*ε_s_−ε_∞_*	*τ* (s)	*β*
0.30	1.7 × 10^−5^	185	3.8 × 10^−2^	0.37
0.41	8.4 × 10^−5^	272	8.0 × 10^−3^	0.49
0.70	5.2 × 10^−3^	570	4.9 × 10^−4^	0.77

## Data Availability

The datasets generated and analyzed during the current study are available from the corresponding authors upon reasonable request.
